# Berberine Improves Benign Prostatic Hyperplasia via Suppression of 5 Alpha Reductase and Extracellular Signal-Regulated Kinase *in Vivo* and *in Vitro*

**DOI:** 10.3389/fphar.2018.00773

**Published:** 2018-07-16

**Authors:** Dong-Hyun Youn, Jinbong Park, Hye-Lin Kim, Yunu Jung, JongWook Kang, Seona Lim, Gahee Song, Hyun Jeong Kwak, Jae-Young Um

**Affiliations:** ^1^Department of Pharmacology and Basic Research Laboratory for Comorbidity Regulation, College of Korean Medicine, Kyung Hee University, Seoul, South Korea; ^2^Department of Science in Korean Medicine, Graduate School, Kyung Hee University, Seoul, South Korea

**Keywords:** berberine, benign prostatic hyperplasia, 5 alpha reductase, androgen receptor, mitogen-activated protein kinase, extracellular signal-regulated kinase

## Abstract

Benign prostate hyperplasia (BPH) is a common disease in elderly men, characterized by proliferated prostate and urinary tract symptoms. The hormonal cascade starting by the action of 5-alpha-reductase (5AR) is known to be one of the pathways responsible for the pathogenesis of BPH. Present investigation evaluated the capacity of berberine (BBR), a nature-derived compound abundant in *Coptis japonica*, in testosterone-induced BPH rats. Experimental BPH was induced by inguinal injection with testosterone propionate (TP) for 4 weeks. BBR or finasteride, a 5AR inhibitor as positive control, was treated for 4 weeks during BPH. BPH induced by TP evoked weight gaining and histological changes of prostate and BBR treatment improved all the detrimental effects not only weight reduction and histological changes but also suppression of prostate-specific antigen (PSA), which is elevated during BPH. Additionally, BBR suppressed TP-associated increase of 5AR, androgen receptor (AR) and steroid coactivator-1 (SRC-1), the key factors in the pathogenesis of BPH. To evaluate the underlying molecular mechanisms responsible for beneficial effects of BBR, we investigated whether these effects were associated with the mitogen-activated protein kinase pathway. BPH induced by TP showed increased phosphorylation of extracellular signal-regulated kinase (ERK), whereas this was suppressed by BBR treatment. On the other hand, c-jun-N-terminal kinase (JNK) and p38 mitogen-activated protein kinase was not changed in BPH rats. In *in vitro* study using RWPE-1 cells, a human prostate epithelial cell line. TP increased cell proliferation and BPH-related key factors such as PSA, AR, and 5AR in RWPE-1 cells, and those factors were significantly decreased in the presence of BBR. Furthermore, these proliferative effects in RWPE-1cells were attenuated by treatment with U0126, an ERK inhibitor, confirming BBR can relieve overgrowth of prostate via ERK-dependent signaling. The cotreatment of U0126 and BBR did not affect the change of 5AR nor proliferation compared with U0126 alone, suggesting that the effect of BBR was dependent on the action of ERK. In conclusion, this study shows that BBR can be used as a therapeutic agent for BPH by controlling hyperplasia of prostate through suppression of ERK mechanism.

## Introduction

Benign prostate hyperplasia (BPH), one of the major urinary disorders of the male elderly, is demonstrated to be histologically distinctive (Lytton et al., 1968). During the process of aging, alteration of major endocrine hormones results in the release of androgen hormones and enlargement of the prostate gland (Arrighi et al., 1991). Generally, the androgen receptor (AR) signaling pathway is known to play a crucial role in the occurrence of BPH (Izumi et al., 2013). Even though the exact mechanism of BPH still needs to be investigated, the close relation between BPH and androgen is able to speculate. It is known that the testosterone produced in the testis spreads to the prostate and is converted to dihydrotestosterone (DHT) by 5-alpha-reductase (5AR), an enzyme involved in steroid metabolism, to regulate the development of BPH (Thigpen et al., 1993; Asada et al., 2001). DHT binds to AR and serves to make AR function as a transcription factor in the nucleus (Heinlein and Chang, 2002b). The oversupply of testosterone leads to an excessive DHT conversion in the prostate via action of 5AR (Clark et al., 2004). This converted DHT has much higher binding ability compared to testosterone (Andriole et al., 2004). In prostate cells, testosterone or DHT combine to AR, leading to increases in transcription of androgen-dependent genes, and as a result, proliferation is stimulated (Roehrborn, 2008). Thus, the pathogenesis of BPH is closely related to the 5AR-AR axis.

The mitogen-activated protein kinase (MAPK) signaling pathways play key roles in the cellular proliferation and growth. Among them, the extracellular signal-regulated kinase (ERK) cascade, which is activated by phosphorylation of MAPK/ERK kinases, is an essential serine/threonine protein kinase for normal cell proliferation and development (Scholl et al., 2007; Frémin et al., 2015). Although the exact role of ERK is yet to be revealed in BPH, Papatsoris and Papavassiliou (2001) suggested the action of MAPK signaling as one of the molecular bases of BPH. Some studies, including our previous report, show that ERK is regulated in the enlarged prostate (Fu et al., 2017; Xu et al., 2017; Youn et al., 2017) or proliferation of prostate stromal cells (Zhang et al., 2008; Youn et al., 2017). Other studies report that the ERK cascade is phosphorylated in reaction to AR, leading to cell cycle progression in prostate cancer (Peterziel et al., 1999; Migliaccio et al., 2000).

Significant enlargement of the prostate impacts the qualities of lives of BPH patients by inducing lower urinary tract symptoms which include urinary intermittency, frequency, straining, urgency, weak stream, incomplete emptying, and nocturia (Barkin, 2011). However, due to the side effects of currently available medications for BPH (Djavan and Marberger, 1999), complementary and alternative herbal medicines has gained recent interest worldwide (Calixto, 2000). Berberine (BBR), a natural compound, is extracted in various herbal medicines such as *Coptis japonica* and *Berberis Koreana* (Kim et al., 2016). Previous studies showed that BBR suppresses both AR signaling pathway (Li et al., 2011; Tian et al., 2016) and ERK-mediated proliferation (Hur and Kim, 2010) in prostate cancer cells. However, its action in BPH has not been confirmed, nevertheless the fact AR and ERK are both important in the process of prostatic hyperplasia. Thereby, we conducted an experiment under the hypothesis that BBR, which has an obvious AR- and ERK-regulating effect, is effective on BPH. The aim of the study was to investigate the effects of BBR on changes in androgen hormone-related factors, MAPK pathway, and eventually on proliferation of prostate cells and tissue in the experimental BPH models *in vivo* and *in vitro*.

## Materials and Methods

### Chemical Reagents

The BBR (≥ 98% pure) was purchased from Sigma Chemicals (St. Louis, MO, United States) and was dissolved in 100% dimethyl sulfoxide (DMSO). TP was provided from Wako pure chemical industries (Osaka, Japan), and Fi (≥ 97% pure) was purchased from Sigma-Aldrich Inc. (St. Louis, MO, United States). Antibodies for AR and SRC1 were purchased from Pierce biotechnology (Rockford, IL, United States), antibody for Ki67 and 5AR were products from Abcam, Inc. (Cambridge, MA, United States). The Antibodies for PSA, p38, and glyceraldehyde-3-phosphate dehydrogenase (GAPDH) was from Santa Cruz Biotechnology (Santa Cruz, CA, United States). p-p38, p-ERK, p-JNK, and JNK were purchased from Cell Signaling Technology (Danvers, MA, United States). Anti-ERK antibody was obtained from Bioworld Technology, Inc. (St. Louis Park, MN, United States). U0126 (≥ 98% pure) was purchased from Merck and Co., Inc. (Kenilworth, NJ, United States). siRNAs for ERK1 and ERK2 were purchased from Santa Cruz Biotechnology.

### Animals

The 12-week-old male Sprague–Dawley (SD) rats with initial body weights of 200–220 g were purchased from the Dae-Han Experimental Animal Center (Dae-Han Biolink, Eumsung, South Korea). The animals were all maintained in conditions in accordance with the regulation issued by the Institutional Review Board of Kyung Hee University [confirmation number: KHUASP(SE)-P-034]. The rats were housed in a pathogen-free room maintained at 23 ± 2°C and a relative humidity of 70% with an alternating 12 h light/dark cycle. Water and standard laboratory diet (CJ Feed Co., Ltd., Seoul, South Korea) were provided *ad libitum*.

### Experimental Procedures

The BPH was induced by a pre-4-week treatment of daily subcutaneous injections of TP (5 mg/kg) at the inguinal region (*n* = 16) as described previously (Youn et al., 2017). Briefly, the rats were divided into four groups with four animals in each group: (1) a normal control group (NC) that received ethanol with corn oil; (2) a BPH group that received TP with corn oil; (3) a positive control group that received finasteride (Fi) (1 mg/kg), a 5ARI which is frequently used as a treatment for BPH, with TP (5 mg/kg); and (4) a group that received BBR (20 mg/kg) with TP (5 mg/kg). BBR and Fi were administered by injection into the inguinal area of animals once daily for 4 weeks, following the pre-4-week BPH inducement by TP injection. After the final treatment, animals were fasted overnight and euthanized using CO_2_. Blood samples were obtained from the caudal vena cava. The blood containing tubes were remained at RT for 2 h and sera were separated by centrifuging at 3000 ×*g* for 20 min in 4°C. The serum was stored at -80°C until further assays. The intact prostate tissue was carefully dissociated and removed, washed with PBS, and then weighed. Relative prostate weight was calculated as the ratio of prostate weight (mg) to body weight (100 g). The prostate tissue was divided in half; one-half was fixed in 10% formalin and embedded in paraffin for histomorphological assays, the other was stored at -80°C for further assays.

### Hematoxylin and Eosin (H&E) Staining and Immunohistochemistry (IHC)

The prostate tissue sections were prepared as described previously (Ko et al., 2016; Park et al., 2017). For H&E staining, the sections were stained in hematoxylin for 5 min, and then washed with water for 5 min. Then the sections were stained in eosin for 30 s, dehydrated, and mounted by routine methods. For immunostaining, sections were incubated in 4°C overnight with a 1:200 dilution of the primary antibody; then incubated at room temperature for 30 min with a 1:500 dilution of the horseradish peroxidase (HRP)-conjugated affinipure Goat anti-rabbit IgG (Jackson ImmunoResearch Laboratories, Inc., West Grove, PA, United States) or HRP-conjugated affinipure Goat anti-mouse IgG (Jackson ImmunoResearch Laboratories, Inc., West Grove, PA, United States). Following the addition of the detection system, the reaction was visualized using diaminobenzidine (DAB) in the presence of hydrogen peroxide. The slides were examined using the Olympus IX71 Research Inverted Phase microscope (Olympus Co., Tokyo, Japan), and the density was measured using ImageJ 1.47v software (National Institute of Health, Bethesda, MD, United States).

### Western Blotting Assay

Protein expression analysis was performed as previously reported (Won et al., 2016). Briefly, homogenized tissues or harvested cells were lysed with ice-cold RIPA buffer, the insoluble materials were removed, and the proteins were separated by 8% sodium dodecyl sulfate-polyacrylamide gel electrophoresis and transferred onto Polyvinylidenedifluoride (PVDF) membranes (Billerica, MA, United States). After blocking with 10 mM Tris, 150 mM NaCl, and 0.05% Tween-20 (TBST) containing 5% skim milk for 1 h at room temperature, the membranes were incubated with the primary antibody at 4°C overnight. The blots were subsequently incubated with HRP-conjugated affinipure Goat anti-rabbit IgG (Jackson ImmunoResearch Laboratories, Inc.) or HRP-conjugated affinipure Goat anti-mouse IgG (Jackson ImmunoResearch Laboratories, Inc.). The protein assay reagent was obtained from Bio-Rad (Hercules, CA, United States). The chemiluminescent intensities of protein signals were quantified using ImageJ 1.47v software (National Institute of Health).

### Cell Culture

The normal human prostatic epithelial cell line RWPE-1 was obtained from the American Type Culture Collection (Manassas, VA, United States). RWPE-1 cells were cultured in Roswell Park Memorial Institute medium (RPMI) (Gibco, Big Cabin, OK, United States) supplemented with 100 mg/ml penicillin/streptomycin (HyClone, Logan, UT, United States) and 10% FBS (Sigma-Aldrich Inc.). After 24 h of incubation, the culture media was replaced with fresh media containing 0.5 μM of TP in order to induce cell proliferation. BBR was supplemented together within TP-containing media. U0126 (10 μM) or siERK1/2 (10 μM) was also supplemented in the TP-containing media.

### MTS Assay

The RWPE-1 cells were seeded (2.5 × 10^4^ cell/well) and incubated in RPMI plus 10% FBS for 24 h. Then, the cells were incubated in fresh media containing various concentrations of BBR for an additional 24 h. Cell viability was monitored using the cell proliferation MTS kit by the Promega Corporation as recommended by the manufacturer. Prior to measuring the viability, the media were removed and replaced with 200 μl of fresh RPMI plus 10% FBS medium and 10 μl of 3-(4,5-dimethylthiazol-2-yl)-5-(3-carboxymethoxyphenyl)-2-(4-sulfophenyl)-2H-tetrazolium (MTS) solution. The cells were then incubated in the incubator for 4 h. The absorbance was measured at 490 nm in a VERSAmax microplate reader (Molecular Devices, Sunnyvale, CA, United States) to determine the formazan concentration, which is proportional to the number of live cells.

### EdU Proliferation Assay

Proliferation of RWPE-1 cells was assessed using the Click-iT EdU Imaging Kit (Invitrogen, Waltham, MA, United States) according to the manufacturer’s protocol. Briefly, cells were plated overnight and labeled with 10 μM EdU for 24 h, and then the cells were transferred into a 6-well plate and fixed with 3.7% formaldehyde for 15 min at RT, washed twice with 3% BSA in PBS. Next, 1 ml of 0.5% Triton X-100 in PBS was added, followed by a 20 min incubation at RT. EdU was detected by staining cells with Click-iT reaction cocktail for 30 min at RT. DNA staining was performed using Alexa Fluor 488 Imaging Kit (Invitrogen, Waltham, MA, United States). Samples were analyzed using iRiS Digital Cell Imaging System (Logos Biosystems, Anyang, South Korea) at 488 nm.

### Immunofluorescence Staining

To evaluate immunofluorescence signals of PSA, AR, 5AR, and Ki67, after treatment with or without BBR, the RWPE-1 cells were washed three times with PBS and fixed with 4% formaldehyde for 15 min at RT. They were permeabilized with PBS containing 0.25% Triton X-100 for 10 min. After that non-specific binding was blocked by incubation with 5% BSA in PBS for 30 min. Then, they were incubated with PSA, AR, 5AR, and Ki67 antibodies in 5% BSA in PBS overnight at 4°C followed by incubation with fluorescent secondary antibody Alexa Fluor 488 and Alexa Fluor 546 for 1 h. Finally, they were incubated with DAPI for 3 min to stain the nuclei. After staining, all the samples were mounted in mounting medium. Images were acquired on a fluorescence microscope (Logos Biosystems, Anyang, South Korea).

### Epididymal Sperm Count Analysis

The 12-week-old SD rats (*n* = 12) were divided randomly into three groups: (1) a vehicle-treated group, (2) Fi (1 mg/kg)-treated group, and (3) a BBR (20 mg/kg)-treated group. The rats were treated with BBR and Fi by injection into the inguinal area of animals once daily for 4 weeks. After the final injection, the rats were fasted overnight and then sacrificed. The cauda epididymis was removed, slashed twice and placed into 4 ml of PBS for 1 h to let sperms diffuse. Then, sperm-containing PBS was mixed with the same amount of methanol, and the epididymal sperm count was done using a hemocytometer under an Olympus IX71 Research Inverted Phase microscope (Olympus Co., Tokyo, Japan). A total of 30 samples from each group was used for evaluation.

### Statistical Analysis

The data values were presented as the mean ± SD. Differences in mean values were analyzed by one-tailed Student’s *t*-test using IBM SPSS Statistics 22 software (International Business Machines Corp., New York, NY, United States). Values with *P* < 0.05 were considered as statistical significance.

## Results

### BBR Attenuates Prostatic Hyperplasia in TP-Induced BPH Rats

Changes in prostate tissue size, prostate weight, and prostate index are shown in **Table 1**. Rats treated with TP showed increase in prostate weight and prostate index by 149.71 and 197.12% compared to the normal control. In comparison with the BPH group, BBR treatment significantly decreased the prostate weight by 18.53% and the prostate index by 17.89%. Fi also decreased the prostate weight and prostate index by 55.83 and 56.53%, respectively. Body weight of rats did not show any difference among TP-treated groups.

**Table 1:** Effect of BBR on body and prostate tissue weights.

	NC	BPH	BBR	Fi
Body weight (g)	466.37 ± 18.22	416.93 ± 13.42	412.70 ± 10.98	422.83 ± 9.16
Prostate weight (mg)	583.33 ± 53.00	1456.67 ± 35.47^#^	1186.67 ± 50.60*	643.33 ± 67.50*
Prostate index	117.72 ± 11.75	349.78 ± 7.57^#^	287.20 ± 4.80*	152.04 ± 14.89*

*Values are mean ± SD of data from three separate experiments. Prostate index was calculated dividing body weight of the rat (100 g) by the prostate weight (mg). ^#^P < 0.05 when compared to NC; ^∗^P < 0.05 when compared to BPH. NC, normal control group; BPH, TP-induced BPH group; BBR, BBR-treated BPH group; Fi, Fi-treated BPH group.*

**FIGURE 1:**
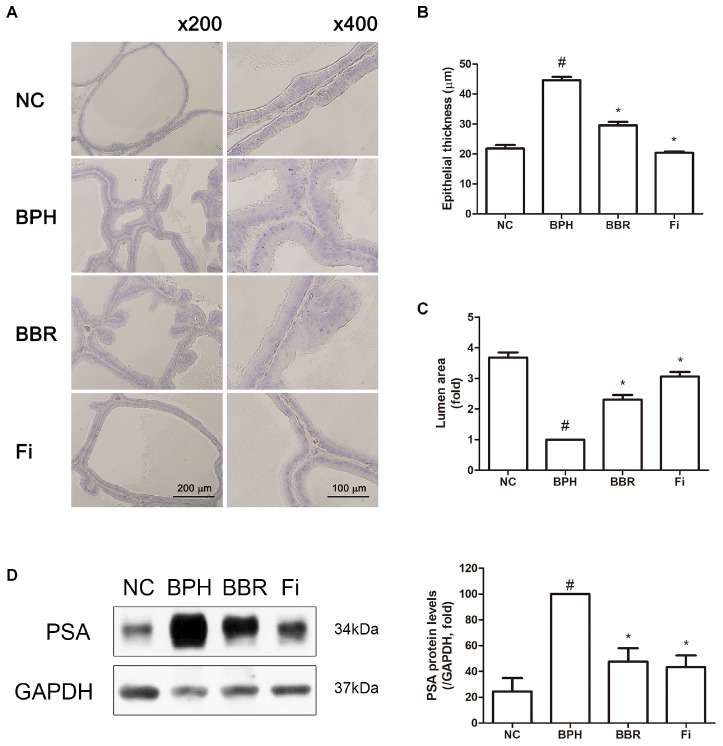
Effect of BBR on histological changes and protein expression of PSA in prostate tissues of TP-induced BPH rats. **(A)** Representative photomicrographs of H&E stained prostate tissues (left panels, magnification ×200; right panels, magnification ×400) are shown. **(B)** The epithelial thickness and **(C)** the relative lumen area of the prostate tissues were measured using ImageJ software. Values are mean ± SD of of ten or more separate measurements. **(D)** The protein expression of PSA was analyzed by a Western blot analysis. Values are mean ± SD of three or more separate measurements. ^#^*P* < 0.05 when compared to NC; ^∗^*P* < 0.05 when compared to BPH. The protein expression differences are normalized to GAPDH. NC, normal control group; BPH, TP-induced BPH group; BBR, BBR-treated BPH group; Fi, finasteride-treated BPH group.

As shown in **Figure 1A**, the structure of prostate tissue, assessed by H&E staining showed various histological changes by TP administration. The epithelium of the prostate gland in BPH group displayed signs of proliferation associating over-formed acinus area with decreased lumen area when compared with control group. However, BBR group and Fi group displayed recovered normal histology without pathological changes in prostate tissues of mice with BPH induced by TP. Furthermore, while the area of lumen was significantly decreased in the TP-administered group compared to the normal control group, BBR-treated group and Fi-treated group had wider lumen than that of the BPH group (**Figures 1B,C**).

### BBR Suppresses PSA Expression in TP-Induced BPH Rats

The increase of PSA is related with the proliferation of prostate cells, thus PSA is clinically used for the diagnosis of BPH (Lazier et al., 2004; Agoulnik et al., 2005; Gravas and Oelke, 2010; Velonas et al., 2013). To determine whether BBR is capable of blocking on the induction of prostate-specific antigen (PSA) during BPH, the effect of BBR was examined in the level of PSA. While TP administration increased the levels of PSA by 4.09-fold, BBR treatment significantly decreased the level of PSA by 47.59% compared to BPH group (4.09-fold increase vs. NC), and similar effects were observed in the Fi-treated positive control group (43.46% reduction vs. BPH group) (**Figure 1D**).

### BBR Regulates 5AR-AR Axis in TP-Induced BPH Rats

**FIGURE 2:**
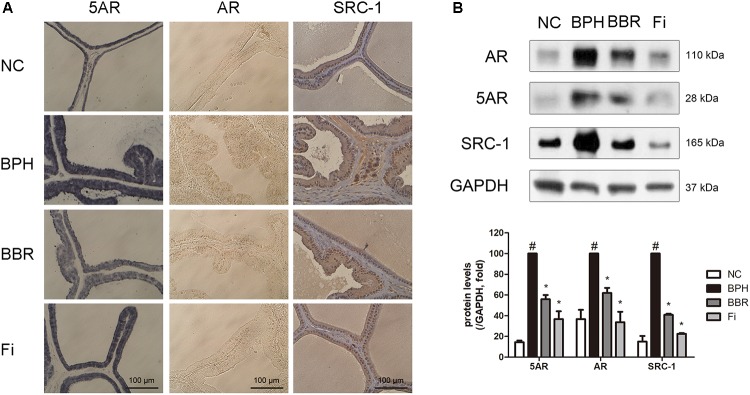
Effect of BBR on expressions of 5AR, AR and its coactivator SRC1 in prostate tissues of TP-induced BPH rats. **(A)** Representative photomicrographs of IHC stained prostate tissues with anti-AR, anti-5AR, and anti-SRC1 antibodies (magnification ×400) are shown. **(B)** The protein expressions of 5AR, AR, and SRC1 were analyzed by a Western blot analysis. Values are mean ± SD of three or more separate measurements. ^#^*P* < 0.05 when compared to NC; ^∗^*P* < 0.05 when compared to BPH. The protein expression differences are normalized to GAPDH. NC, normal control group; BPH, TP-induced BPH group; BBR, BBR-treated BPH group; Fi, finasteride-treated BPH group.

The prostatic hyperplasia was examined by IHC staining using antibodies against 5AR, AR, and SRC-1. 5AR is the major factor that converts testosterone into DHT. DHT then binds to AR, and AR-coactivators such as SRC-1 are combined as well with DHT in the hyperplasia process of the prostate (Thigpen et al., 1993; Asada et al., 2001; Agoulnik et al., 2005). As shown in **Figure 2A**, these aforementioned factors in were increased higher in the TP-treated BPH group than in the normal control group. Elevated protein expressions of the three 5AR-related factors, 5AR-2, AR, and SRC-1 were confirmed in the BPH group. Remarkably, significant reduction in the protein expression of such factors was shown in BBR-treated rats (**Figure 2B**).

### BBR Suppresses TP-Induced Increase of ERK Phosphorylation in TP-Induced BPH Rats

**FIGURE 3:**
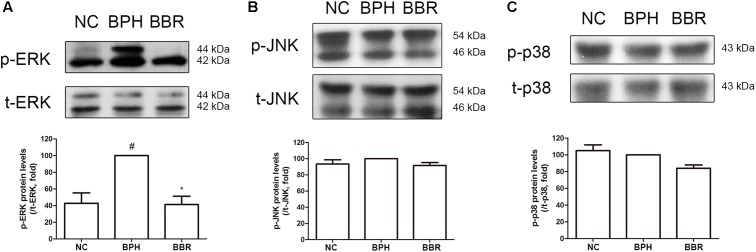
Effect of BBR on MAPK expressions in prostate tissues of TP-induced BPH rats. The phosphorylation levels of **(A)** ERK, **(B)** JNK, and **(C)** p38 were analyzed by a Western blot analysis. Values are mean ± SD of three or more separate measurements. ^#^*P* < 0.05 when compared to NC; ^∗^*P* < 0.05 when compared to BPH. The protein expressions differences of p-ERK, p-JNK, and p-p38 were normalized to total ERK, total JNK, and total p38, respectively. NC, normal control group; BPH, TP-induced BPH group; BBR, BBR-treated BPH group.

The MAPKs pathway is well known to be involved in cell proliferation and apoptosis (Seger and Krebs, 1995). Thus, we examined whether the role of MAPKs is also important in the mechanism of BBR-mediated beneficial effects in BPH. The phosphorylation of ERK, which is a major proliferative factor in the cell, dramatically increased in the BPH group compared to normal control group. BBR treatment abrogated the protein expression of p-ERK to the similar level as in the normal control group as statistical power (**Figure 3A**). Neither p-JNK nor p-p38 elicited an induction on BPH group (**Figures 3B,C**). These observations imply that beneficial effect of BBR against TP-induced BPH indeed appear to be mediated through the ERK pathway but not the JNK or p38 cascade.

### BBR Regulates BPH-Related Factors in TP-Treated Proliferated RWPE-1 Cells

**FIGURE 4:**
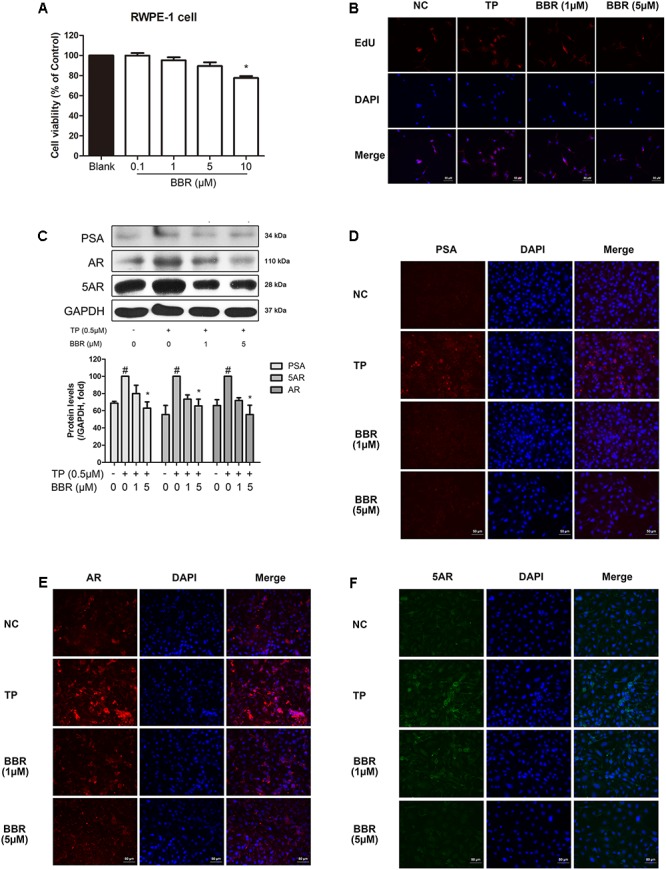
Effect of BBR on BPH-related factors in TP-treated RWPE-1 cells. **(A)** Cell viability was assessed by an MTS assay. Values are mean ± SD of 10 or more separate experiments ^∗^*P* < 0.05 when compared to TP. **(B)** Representative photomicrographs of EdU assay of TP-treated RWPE-1 cells are shown. **(C)** The relative protein expressions of PSA, AR, and 5AR were analyzed by a Western blot analysis. Values are mean ± SD of three or more separate measurements. ^#^*P* < 0.05 when compared to NC; ^∗^*P* < 0.05 when compared to BPH. The protein expressions differences were normalized to GAPDH. IF staining of **(D)** PSA (red), **(E)** AR (red), and **(F)** 5AR (green) merged with DAPI (blue) was performed. NC, normal control group; TP, TP-treated group; BBR, BBR-treated group.

In order to confirm the mechanism of BPH-improving effect of BBR, we established an BPH mimicking *in vitro* model using RWPE-1 cells, a normal prostate epithelial cell line (Choi et al., 2016) by treating 0.5 μM TP (Ren et al., 2010). In **Figure 4A**, cell viability was analyzed using an MTS assay and no obvious cytotoxicity was observed except 10 μM BBR in TP-induced RWPE-1 cells. TP effectively induced proliferation of RWPE-1 cells, and BBR treatment attenuated their proliferation in a concentration-dependent manner (**Figure 4B**). BBR at 5 μM significantly suppressed the TP-induced BPH-related factors such as PSA, AR, and 5AR when evaluated by immunofluorescence staining and Western blot analysis (**Figures 4D–F**).

**FIGURE 5:**
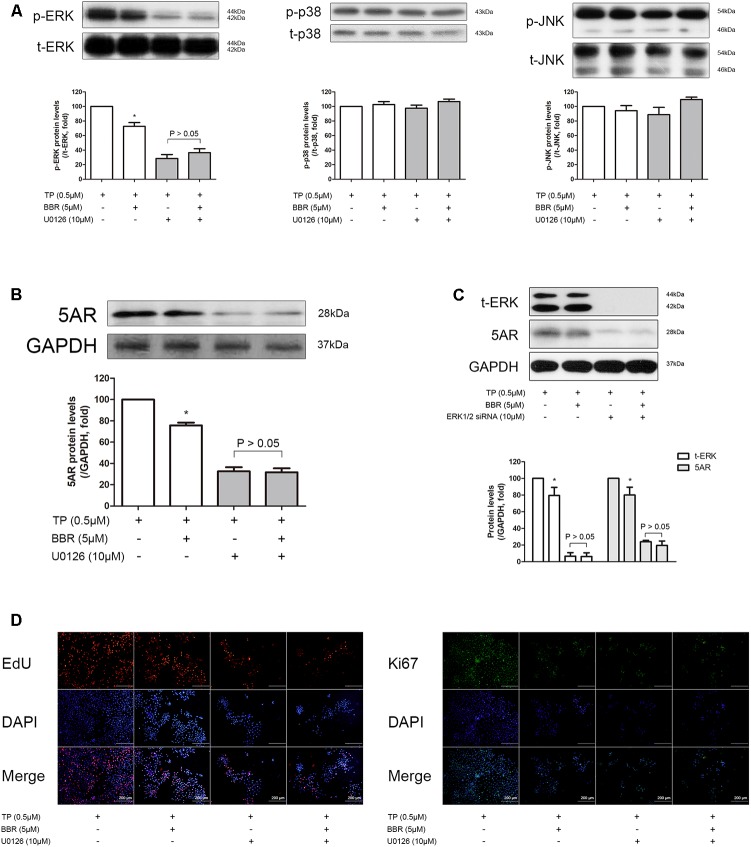
Effect of BBR on BPH-related factors after ERK inhibition in TP-treated RWPE-1 cells. The relative protein expressions of **(A)** p-ERK, p-JNk, p-p38 and **(B)** 5AR after U0126 co-treatment were analyzed by a Western blot analysis. **(C)** Relative protein expressions of t-ERK and 5AR after siERK1/2 co-treatment were analyzed by a Western blot analysis. Values are mean ± SD of three or more separate experiments. ^∗^*P* < 0.05 when compared to TP. The protein expressions differences of 5AR were normalized to GAPDH. The protein expressions differences of p-ERK, p-JNK, and p-p38 were normalized to total ERK, total JNK, and total p38, respectively. **(D)** Representative photomicrographs of EdU assay of ERK-inhibited RWPE-1 cells are shown. IF analysis of Ki67 (green) merged with DAPI (blue) was performed. NC, normal control group; TP, TP-treated group; BBR, BBR-treated group.

In addition, BBR, in consistent with the *in vivo* results, reduced the increased p-ERK levels, but not as much as U0126, a widely used ERK inhibitor (Fukazawa et al., 2002) in TP-treated RWPE-1 cells (**Figure 5A**, left panel). p-JNK or p-p38 was not affected by TP or BBR treatment (**Figure 5A**, middle and right panel). Furthermore, both BBR and U0126 suppressed 5AR expression, and the inhibitory effect of BBR on 5AR expression was nullified by co-treatment of BBR and U0126 (**Figure 5B**). Similar results were shown when we blocked ERK by co-treating TP-induced RWPE-1 cells with siERK1/2 (**Figure 5C**). Concomitantly, we evaluated the anti-proliferative effect of BBR in association with ERK activation by EdU and Ki67 staining. Inhibition of ERK phosphorylation by U0126 treatment resulted in abolished anti-proliferative effect of BBR (**Figure 5D**). These results indicate the possibility of ERK involvement in the pathology of prostate hyperplasia, suggesting the role of BBR as an ERK inhibitor as well.

### BBR Treatment Does Not Affect Spermatogenesis *in Vivo*

**FIGURE 6:**
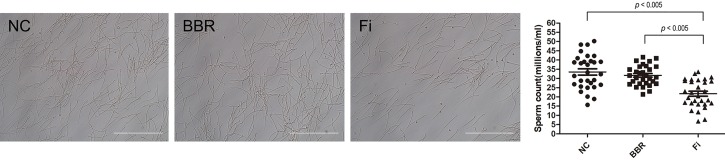
Effect of Fi and BBR on epididymal sperm numbers. The sperm numbers were evaluated after daily subcutaneous injection with Fi (1 mg/kg) or BBR (20 mg/kg) for 4 weeks. Values are mean ± SD of 30 separate measurements. NC, normal control group; Fi, finasteride-treated group; BBR, BBR-treated group.

One of the fatal side effects of 5ARI such as Fi is reduced sperm numbers (Amory et al., 2007; Samplaski et al., 2013). Reduced sperm count is also observed in Fi-treated rats as well as human. Serga (2009) has reported high concentration of Fi treatment results in decreased epididymal sperm number in SD rats. Based on this study, we evaluated the sperm numbers in the epididymis of vehicle-, Fi-, and BBR-treated rats. As shown in **Figure 6**, Fi treatment resulted in a significant reduce of sperm numbers compared to untreated rats, while BBR treatment did not affect the number of sperms.

## Discussion

The LUTS is a disease that affects the quality of life in elderly men, among which BPH possesses the largest proportion (Donnell, 2011). Pathologic enlargement of the prostate (≥ 30 ml vs. normal prostate of 15–30 ml) results in urethral compression, leading to disturbance in urinal flow. This blockage of urine flow is called bladder outlet obstruction (BOO). Continuous BOO can eventually develop into LUTS, and therefore, BPH is a considerable burden on aging men (Chughtai et al., 2016).

According to a preliminary research, 70% of men over the age of 70 suffer from BPH (Berry et al., 1984). However, the pathophysiology of BPH development is not fully understood, due to its complication. Several risk factors such as metabolic syndrome, diet, age are related to the development of BPH. The pathogenesis of BPH is also not clear. Inflammation is considered as a crucial mechanism of pathologic prostate growth. Studies report increased cytokines and inflammatory cell infiltration in BPH tissues (Kramer et al., 2002; Steiner et al., 2003). The smooth muscle tone in prostate tissues is also related. As a substantial proportion of the prostate gland is composed of smooth muscles, the contractile property of these muscles can impact the urinal flow (Shapiro et al., 1992). However, the sex hormones, both androgens and estrogens, are considered to be the most important factor in the pathogenesis of BPH. The 5AR-converted DHT acts on prostate cells to regulate the homeostasis between apoptosis and proliferation, leading to the overgrowth of prostate cells (Chughtai et al., 2016).

As prostatic hyperplasia develops, its related factors such as PSA and AR are known to be increased (Henttu and Vihko, 1993; Culig et al., 1996). Both 5AR and AR are crucial factors in development of BPH, however, ERK activation is also necessary and sufficient for the growth and development of the prostate (Papatsoris and Papavassiliou, 2001). This role of ERK as a stimulator of prostate proliferation is known to be independent of androgen signaling (Gao et al., 2006). Supporting these reports, our previous study also demonstrated that increased ERK activation occurs in BPH (Youn et al., 2017).

Alpha-blockers and 5AR inhibitors (5ARIs) are currently the only two therapeutic options for BPH treatment. Alpha-blockers are antagonists acting against α1-adrenergic receptors, which are commonly chosen for initial therapy of BPH (Gilbert et al., 2006; Hutchison et al., 2007; Roehrborn et al., 2007). However, despite its effectiveness in relaxing smooth muscle of prostate and thereby resolving of blocked urinal flux, they cannot decrease the size of the prostate, and furthermore frequently show side effects including hypotension and headaches (Djavan and Marberger, 1999). Regarding the action of 5ARIs on BPH, the beneficial actions of 5ARI on the shrinkage of the enlarged prostate tissue appeared to be due to the suppression of 5AR activity (Roehrborn et al., 2004). Although the efficacious effects of 5ARIs are well recognized, serious side effects like loss of libido and erectile dysfunction are displayed commonly (Gormley et al., 1992). Due to such adverse effects of available treatments, exploring naturally occurring compounds beneficial for BPH patients has gained recent interest (Agbabiaka et al., 2009; Rick et al., 2012). Indeed, in our findings, BBR, the major compound of *Coptis japonica* plays an important role in the regulation of BPH related factors via ERK signaling and 5AR-AR axis, without displaying side effects as Fi (**Figure 6**).

Since Kato et al. (1965) has reported pathologic growth of prostate tissue in TP-treated rats, this experimental model has been widely used by researchers for BPH research (Maggi et al., 1989; Shin et al., 2012; Li et al., 2018). Based on this TP-induced BPH rat model, we attempted to elucidate the effect of BBR. Our results show 20 mg/kg/day of BBR treatment for 4 weeks significantly reduced both size and weight of the prostate tissue compared to those of the TP-induced BPH control group. In particular, its efficacy under BPH conditions was attributed to the amelioration of both prostatic stromal and grandular components, which are known to be increase in BPH condition (Gao et al., 2006). In addition, BBR reduced atrophied acinar gland in the prostatic epithelial cells, suggesting beneficial effects of BBR on TP-induced prostatic hyperplasia (**Figure 1** and **Table 1**).

The PSA, a protein that is specifically expressed in the prostate gland, is an indicator to identify when prostate cells are over-proliferated in diseases such as BPH or prostate cancer (Velonas et al., 2013). In accordance with this, PSA protein was overexpressed in TP-induced BPH rats, implying that their overexpression is associated with proliferation of prostate cells. Although the cause of prostate enlargement is not fully known, the importance of the action of 5AR-AR axis is well established (Aggarwal et al., 2010). Our findings show BBR treatment can suppress the PSA protein expression in the TP-induced BPH model. BBR treatment also decreased 5AR, the first trigger for prostate enlargement, and AR, the most important receptor related in BPH pathogenesis. Moreover, BBR action on BPH was found to reduce expression of SRC-1, a coactivator of AR-complex (Heinlein and Chang, 2002a). Through our results, the protective actions of BBR on BPH appear to be dependent of 5AR-AR activity reduction (**Figures 1, 2**).

The MAPKs – ERK, JNK, and p38 – are a class of proline-directed serine/threonine kinases, which are involved in the cell cycle, proliferation, and apoptosis (Seger and Krebs, 1995; Rauch et al., 2016). Among these, the ERK cascade is stimulated by growth factors produced in prostatic cells (Manimaran et al., 2016). It has been also reported that activated ERK1/2 signaling increase cancer cell survival and chemoresistance (Tran et al., 2001; Llorens et al., 2004). Although several other studies also showed that the MAPK pathway is closely related to cell proliferation in prostate cancer (Papatsoris et al., 2007; Rybak et al., 2015), the exact role of MAPK signaling pathway in BPH is yet to be cleared. Especially, ERK signaling is a considerable interest in BPH because our and other previous studies showed that the ERK signaling is activated in BPH conditions *in vivo* and *in vitro* (Zhang et al., 2008; Fu et al., 2017; Xu et al., 2017; Youn et al., 2017). In consistent with such reports, our findings indicate that ERK is dramatically activated under BPH conditions and subsequently returned to normal levels by BBR treatment, but phosphorylation levels of JNK and p38 were not significantly affected in BPH-induced rats (**Figure 3**). These report strongly support the notion that downregulation of ERK mediates protective action of BBR in BPH.

The RWPE-1 cells are epithelial cells derived from normal human prostate (Bello et al., 1997) and it is able to mimic BPH conditions *in vitro* by TP treatment. Therefore, this model is well suited for the analysis of BBR action, thus helping to understand involved mechanisms in detail. Based on our results, RWPE-1 cells treated with TP induce proliferation. In parallel, the inhibitory effect of BBR on TP-induced prostatic cell proliferation was also observed by the same experimental method. As expected, protein levels of PSA, AR, and 5AR were all significantly elevated in TP-treated RWPE-1 cells, and then was suppressed to normal levels by BBR (**Figure 4**). Treatment with BBR in TP-treated RWPE-1 cells also significantly reduced the phosphorylation level of ERK. Furthermore, pharmacological inhibition of ERK using its specific inhibitor U0126 abolished ERK phosphorylation in TP-treated cells, which supports and reflects our *in vivo* results, suggesting that ERK pathway plays a crucial role in the protective mechanism of BBR (**Figure 5**).

## Conclusion

In this study, we demonstrated the effects of BBR on prostatic proliferation in TP-induced BPH models. The weight of prostate tissue and prostate index was significantly increased by TP treatment, and those factors were reduced by BBR treatment. BBR treatment also restored epithelial thickness and lumen size similar to normal prostate groups, and acinar gland was also decreased. In addition, BBR treatment significantly reduced PSA expression which was increased by TP administration. TP treatment also elevated expressions of the functional enzyme 5AR, the functional receptor AR, and its coactivator SRC-1. We confirmed the levels of these factors were significantly inhibited by BBR. Then, we observed phosphorylation of ERK, but not JNK and p38, was increased by TP-injection, and the elevated p-ERK was suppressed by BBR treatment. Next, we treated the human prostate epithelial cell line RWPE-1 cells with TP, and investigated the factors related in BPH to establish an *in vitro* model for BPH study. As expected, TP administration increased cell proliferation, expressions of PSA, 5AR, and AR. BBR treatment successfully inhibited the TP-induced changes in RWPE-1 cells as well, subsequently to the *in vivo* results. Furthermore, we evaluated the phosphorylation of ERK in the BPH-like *in vitro* model, and investigated its role in the effect of BBR by co-treating TP-induced proliferated RWPE-1 cells with the ERK inhibitor U0126. Our results described above suggest the beneficial effects of BBR in BPH, leading to the possibility of BBR as a therapeutic agent with less side effects for BPH.

## Author Contributions

D-HY performed the *in vitro* experiments. D-HY, YJ, JWK, SL, GS, and H-LK performed the in vitro experiments. HJK provided the technical and material support. D-HY collected the data. D-HY, JP, and J-YU wrote the manuscript. J-YU designed and supervised the study.

## Conflict of Interest Statement

The authors declare that the research was conducted in the absence of any commercial or financial relationships that could be construed as a potential conflict of interest.
