# Corrigendum: Berberine Improves Benign Prostatic Hyperplasia via Suppression of 5 Alpha Reductase and Extracellular Signal-Regulated Kinase *in Vivo* and *in Vitro*

**DOI:** 10.3389/fphar.2019.00541

**Published:** 2019-05-22

**Authors:** Dong-Hyun Youn, Jinbong Park, Hye-Lin Kim, Yunu Jung, JongWook Kang, Seona Lim, Gahee Song, Hyun Jeong Kwak, Jae-Young Um

**Affiliations:** ^1^Department of Pharmacology and Basic Research Laboratory for Comorbidity Regulation, College of Korean Medicine, Kyung Hee University, Seoul, South Korea; ^2^Department of Science in Korean Medicine, Graduate School, Kyung Hee University, Seoul, South Korea

**Keywords:** berberine, benign prostatic hyperplasia, 5 alpha reductase, androgen receptor, mitogen-activated protein kinase, extracellular signal-regulated kinase

In the original article, there was a mistake in the legend for Figure 1 as published. The magnifications of Figure 1A were stated as ×100 and × 400, when they were actually × 200 and × 400. The correct legend appears below.

**Figure 1:**
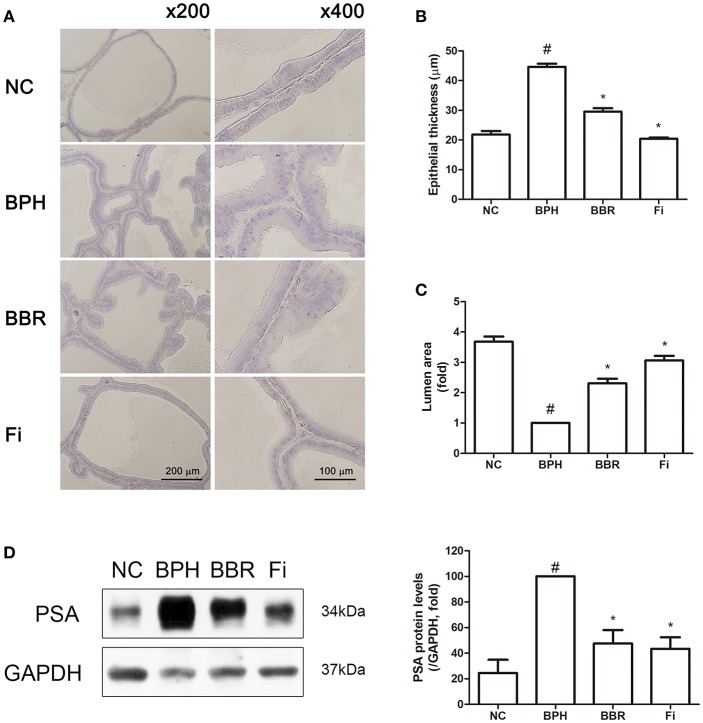
Effect of BBR on histological changes and protein expression of PSA in prostate tissues of TP-induced BPH rats. **(A)** Representative photomicrographs of H&E stained prostate tissues (left panels, magnification ×200; right panels, magnification ×400) are shown. **(B)** The epithelial thickness and **(C)** the relative lumen area of the prostate tissues were measured using Image J software. Values are mean ± S.D. of ten or more separate measurements. **(D)** The protein expression of PSA was analyzed by a western blot analysis. Values are mean ± S.D. of three or more separate measurements. ^#^*P* < 0.05 when compared to NC; ^*^P < 0.05 when compared to BPH. The protein expression differences are normalized to GAPDH. NC, normal control group; BPH, TP-induced BPH group; BBR, BBR-treated BPH group; Fi, finasteride-treated BPH group.

In the original article, there was a mistake in Figure 1 as published. The wrong photomicrographs of prostate tissues were presented by mistake. The corrected Figure 1 appears below. The authors apologize for this error and state that this does not change the scientific conclusions of the article in any way. The original article has been updated.

